# Strain Transfer of Fiber Bragg Grating Sensor Externally Bonded to FRP Strip for Structural Monitoring after Reinforcement

**DOI:** 10.3390/ma14164382

**Published:** 2021-08-05

**Authors:** Soo-Yeon Seo, Jeong-Hun Park, Hyun-Do Yun, Kang-Su Kim, Gun-Cheol Lee, Seongwon Hong

**Affiliations:** 1 Department of Architectural Engineering, Korea National University of Transportation, Chungju 27469, Korea; gclee@ut.ac.kr; 2 M.C.S. Structural Engineering Co. Ltd., Seoul 05836, Korea; pjh4198@naver.com; 3 Department of Architectural Engineering, Chungnam National University, Daejeon 34143, Korea; wiseroad@cnu.ac.kr; 4 Department of Architectural Engineering, University of Seoul, Seoul 02054, Korea; kangkim@uos.ac.kr; 5 Department of Safety Engineering, Korea National University of Transportation, Chungju 27469, Korea; shong@ut.ac.kr

**Keywords:** fiber-reinforced polymer strip, monitoring, optical fiber sensor, fiber Bragg grating, epoxy resin, adhesion length, sensing performance

## Abstract

To date, a method of attaching a FRP (fiber-reinforced polymer) to concrete members with epoxy has been widely applied to increase the strength of the member. However, there are cases in which the adhesion of the epoxy deteriorates over time and the reinforcing effect of the FRP is gradually lost. Therefore, monitoring whether or not the reinforcing effect is properly maintained is needed in order to prevent a decrease in the structural performance of the member improved by FRP reinforcement. In this regard, this study examines FRP with OF (optical fiber) sensors to monitor the reinforcing effect of FRP in concrete structural members. In particular, this paper seeks to determine an appropriate adhesion length when FBG (fiber Bragg grating) based OF sensors are externally bonded to FRP strips with epoxy resin. To this end, a tensile test was carried out to evaluate the sensing performance according to the adhesion length. In addition, an analytical approach was performed and the result were compared with test result. The results of the experimental and analytical studies showed that the strain generated in the FRP is sufficiently transferred to the OF if the total adhesion length of it is 40 mm or more in consideration of the error in the epoxy thickness.

## 1. Introduction

In order to identify the structural state of a building during a service load duration, SHM (structural health monitoring) is needed to measure and manage the magnitude of stress or structural deformation in response to the applied load [1,2,3]. The OF (optical fiber) sensor introduced by Hill [4] is one of the measurement sensors that have recently begun to attract the attention of SHM. OF sensors have advantages over conventional electrical resistance measuring instruments because they do not generate noise in measured values in terms of not being affected by electromagnetic waves, long-term measurement is possible, and excellent corrosion resistance. In addition, it is capable of making multiple measurements with one line and networking [5,6,7]. The FBG (fiber Bragg grating) based OF sensor is already known as a highly reliable and stable measurement sensor capable of monitoring structural behaviors [8]. Consequently, the FBG-based OF sensor has been widely applied to nondestructive evaluation technologies for measuring changes due to temperature and deformation of structural members [9].

Recently, a method of improving the strength of a member by bonding FRP (fiber-reinforced polymer) with epoxy to reinforce the member has been extensively applied to reinforced concrete structures [10,11,12,13,14]. For such FRP reinforcement, it is very important that the reinforcing effect continues during the period of use of the structure. In this manner, the stress or strain of the FRP reinforcement needs to be monitored. When monitoring the FRP reinforcement, it should be observed (1) whether the stress is maintained within the performance limit value of the member and (2) whether the reinforcing effect of FRP reinforcement disappear. The former is a case where the load acting on the member increases, and the latter is a case where the reinforcing effect of the constructed FRP is reduced. If the adhesion function of the epoxy is reduced due to construction mistakes or environmental problems, the reinforcing effect of FRP may gradually disappear. In that case, it is necessary to monitor whether or not the reinforcing effect is properly maintained in order to prevent the problem of a rapid decrease in the structural performance of the member improved by FRP reinforcement. As a method of measurement, an electric strain gauge (ESG) can be used. However, ESG is considered inappropriate for long-term measurements because it is highly subject to electrical and external influences. In this respect, it is desirable to use OF with a high degree of reliability and stability. To continuously monitor the reinforcing effect of FRP in reinforced concrete structural members, research on technologies to apply the FBG-OF sensor is in progress.

Regarding the reinforcement of concrete members using FRP combined with the OF sensor, the core research is focused on the combination of OF sensor and FRP and the monitoring performance after the reinforcement of the concrete member. Relevant studies have been carried out in this regard. Typical examples of such studies include research on strain transfer performance according to the modulus of OF coating and the thickness of an adhesive that attaches the OF sensor to the FRP, the host material [15], and research on the debonding mechanism of FRP by using the embedded optical fiber sensor at the interface between concrete cracks and FRP [16]. In addition, an experimental study was conducted to evaluate the monitoring accuracy of an FBG-OF sensor embedded in the carbon fiber reinforced polymer (carbon FRP) surface, and the findings of the study showed that the embedded FBG-OF sensor can effectively and reliably monitor the inner conditions of composite materials [17]. Meanwhile, to develop a smart CFRP system, Schaller et al. [18] investigated a method of bonding the FBG-OF to the CFRP and conducted four points bending tests on beams and tests on wrapped columns, thereby confirming effective monitoring. Sundaram [19] installed FBG-OF sensors between concrete and FRP under bending stress and used them to effectively identify the propagation of debonding of FRP. In relation to the NSM (near-surface mounted) FRP reinforcement, Wang [20] fabricated smart carbon fiber reinforced polymer (Glass FRP) bars with the FBG-OF sensor embedded in the GFRP bars and evaluated the NSM retrofit and strain monitoring effects using the smart GFRP bars on the basis of experimental investigation on the tensile, bond and flexural behaviors of beams. In order to evaluate the sensing capability of FBG-OF through the bending test as mentioned above, the optimal attachment length under tensile stress must first be identified. Accordingly, many studies have evaluated the sensing performance of FBG-OF through tensile tests [21,22,23,24,25]. As a recent study in this regard, Motwani [24] performed uniaxial tensile test and analysis about a OF sensor mounted on a carbon-fiber-reinforced polyphenylene sulphide specimen using two adhesive types, i.e., a cyanoacrylateand and epoxy-resin to evaluate the sensing capacity. Seo et al. [25] suggested a proper bond length of adhesive for combining FBG-OF between two CFRP strips for NSM reinforcing through a tensile test and analytical approach when using epoxy as adhesive. The method used for evaluation in these experiments was to attach the ESG together and compare it with the OF.

Epoxy resins are mainly used to bond the OF sensor to the surface of the FRP. The main variables that can affect the sensing performance of the FBG-OF sensor bonded to the FRP include the thickness of the adhesive and the elastic modulus of the coating material surrounding the sensor. It is known that when the thickness of the adhesive increases, the deformation of the OF sensor is smaller than that of the host material [15,25]. This in turn suggests that there may be a difference in the sensing capacity depending on the method of attaching the OF sensor to the FRP and bonding materials.

This study aims to determine an appropriate adhesion length when the FBG-OF sensor is externally bonded to the FRP strip with the epoxy resin under working load condition. For this purpose, the sensing performance according to the adhesion length was evaluated through a tensile test, and a theoretical approach was taken to investigate the effects of the adhesion length.

## 2. FRP Strip with Externally Bonded FBG-OF

**Figure 1:**
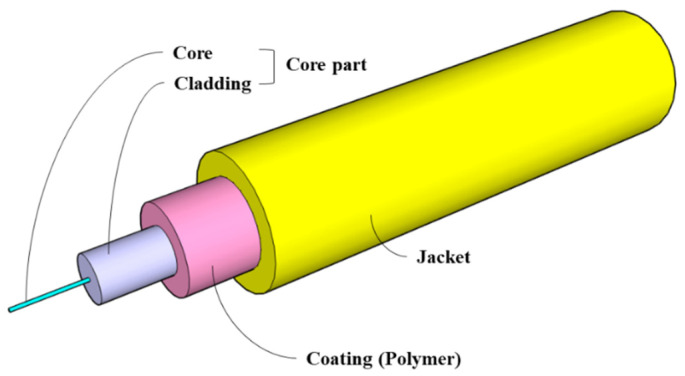
Configuration of optical fiber.

**Figure 2:**
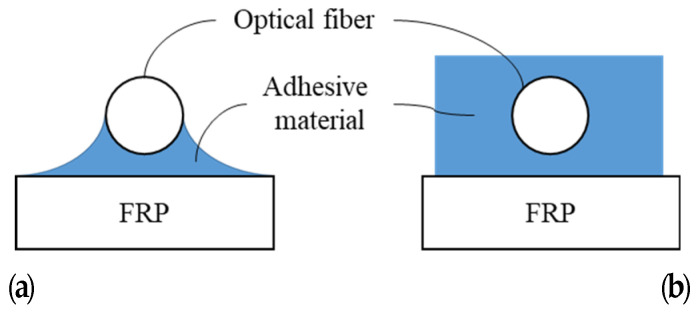
Bonding concept of FBG-OF to the surface of FRP strip: (**a**) attaching lower part of OF by adhesive; (**b**) covering OF with adhesive.

**Figure 3:**
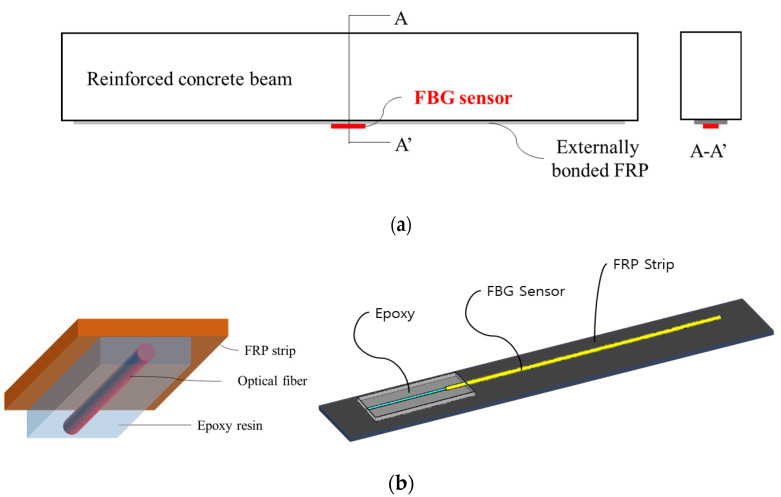
Combining concept of the FBG-OF sensor with FRP strip for external reinforcement of reinforced concrete member: (**a**) external reinforcement of reinforced concrete beams with FRP strip; (**b**) detail of bonded OF sensor.

Typical FBG-OF is composed of the core, cladding, coating and jacket as shown in Figure 1, and the core and cladding constitute a core part. The jacket plays the role of preventing the brittle failure of OF due to an impact or bend. In order to give the sensing function to OF, the FBG sensor is usually formed on the core, and this part is coated. The coated part is then directly attached to the host material to fix the OF with the FBG sensor to it. The method of externally bonding the OF with the FBG sensor to the FRP, which is the host material, is to apply an adhesive thinly on the host material and then place the OF on it as shown in Figure 2a or to cover the entire OF with the adhesive as shown in Figure 2b. When the fabricated FRP strip is used for external retrofit, it is attached to the surface of the concrete member as shown in section A-A’ of Figure 3a. In this paper, experimental and theoretical studies are conducted to determine the method of attaching the FBG-OF to the FRP and the optimal adhesion length under these conditions.

## 3. Tensile Test of FRP Strip with Externally Bonded FBG-OF Sensor

### 3.1. Test Plan

The efficiency of transferring the strain information generated in the FRP to the OF sensor varies depending on the shear lag phenomenon that occurs between the core part (including core and cladding) and coating and the coating and epoxy resin, and the degree of slip at the interface. This is closely related to the properties of the adhesive used for bonding the OF to the FRP, bonding method and adhesion length.

**Figure 4:**
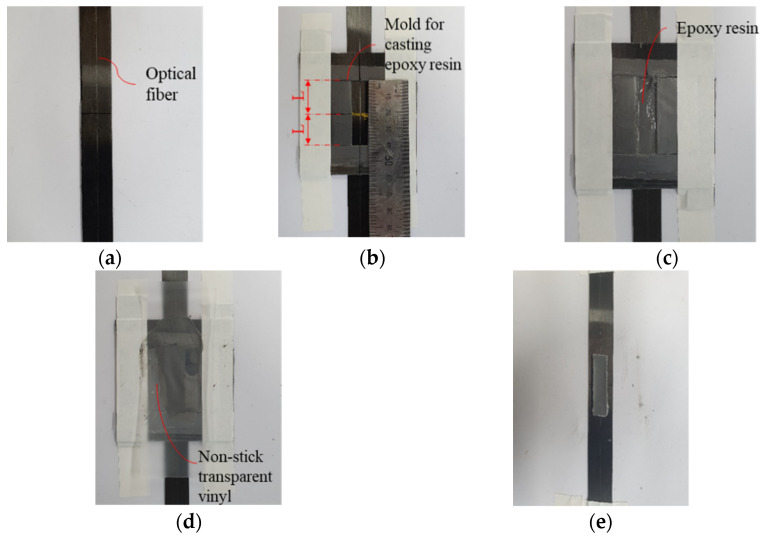
Combining process of OF on FRP strip: (**a**) installation of OF on FRP; (**b**) installation of mold; (**c**) filling epoxy resin; (**d**) flattening the epoxy resin; (**e**) after curing the epoxy resin.

In order to examine the sensing performance according to the adhesion length when the OF sensor is bonded to the surface of the FRP strip with the epoxy resign, a tensile test was performed after combining the OF sensor to the FRP strip with the adhesion length as a variable. Table 1 shows a list of test specimens. In Table 1, the adhesive length, L, is the length in one direction based on the center of the FBG-OF sensor and is 1/2 of the total adhesion length. Figure 4 shows the process of fixing OF to the surface of the FRP strip. First, clean the surface of the FRP strip with acetone, place OF on the FRP strip taut as shown in (a), and fix it with tape about 100mm away from the center in both directions. Next, a pair of molds for epoxy resin filling are fixed on the FRP strip with tape as shown in (b). At this time, the OF is placed between these molds. As shown in (c), the epoxy resin is filled inside the molds and cured by pressing flat with nonstick transparent vinyl as shown in (d). After sufficiently hardening, when the nonstick transparent vinyl and mold are removed, the FRP strip with an OF sensor attached to the surface can be obtained, as shown in (e). The epoxy adhesive has a width of 10 mm and a thickness of 2 mm. ESGs were attached to the same position on the back to which the OF was bonded for each specimen. Tensile tests were performed three times with one specimen for each adhesion length. Epoxy may be affected by temperature during curing [26]. In order to minimize the effect of temperature, the temperature at the time of manufacturing the specimen and during the experiment was kept as constant as possible. The range of temperature was 22 ± 2 °C, the recommendation of the manufacturer.

**Figure 5:**
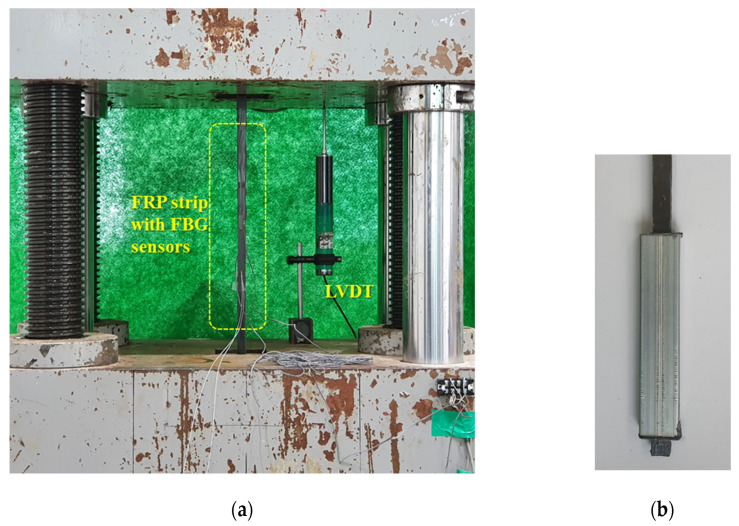
Setup for tensile test: (**a**) test setup; (**b**) end reinforcement.

Figure 5 shows the tensile test setup, where the end portion of the FRP strip was reinforced with an epoxy-filled square steel box to introduce tensile force into the specimen with reference to ASTM D3039 [26]. The specifications and mechanical properties of the OF are presented in Table 2. The length of the FBG was 10 mm. Micron optics si155 was used as the interrogator. The reflectance ratio was more than 70% and the resolution was 1 picometer. The specifications and mechanical properties of the FRP strip and epoxy resin are summarized in Table 3 and Table 4. The tensile force applied to the specimen was 900 MPa, about one third of the tensile strength of the FRP strip. The load was removed after the target strength was reached, and then applied again. In this way, the experiment was conducted by repeating the process three times for each specimen.

### 3.2. Test Result

**Figure 6:**
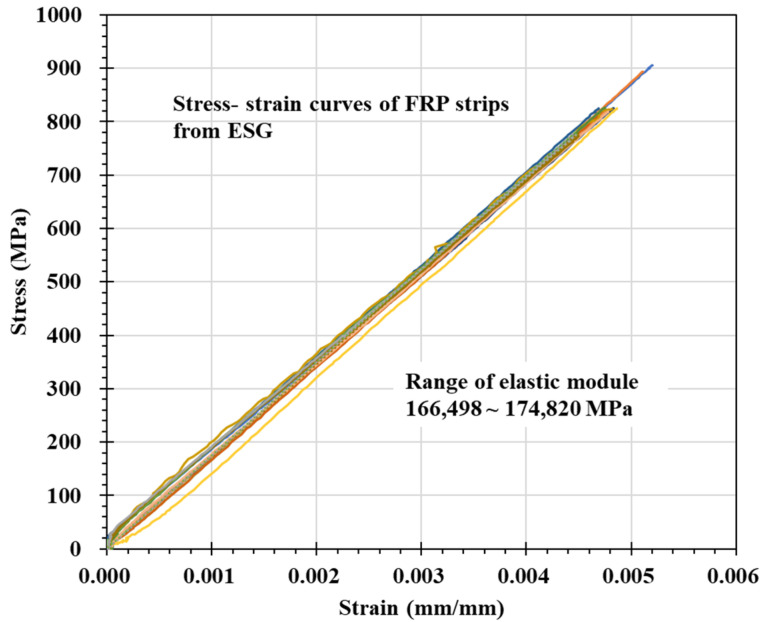
Stress-strain relationship of FRP strip.

As a result of the experiment, the strain increased linearly as the applied load increased. Figure 6 shows the relationship between ESG strain and stress in all experiments. At this time, the stress is obtained by dividing the load by the cross-sectional area of the FRP strip. The cross-sectional area of each specimen was calculated considering the width and thickness measured for each specimen. In all specimens, the stress-strain relationship maintained a linear relationship, and the elastic modulus ranged from 166,498 to 174,820 MPa. This value is somewhat higher than the FRP strip elastic modulus of 165,000 MPa provided by the manufacturer.

**Figure 7:**
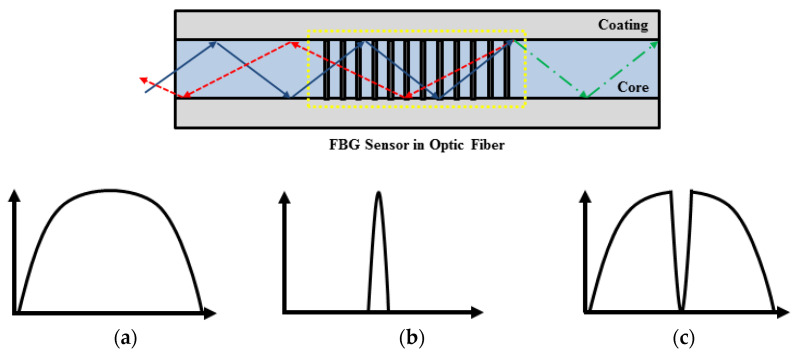
Reflection of wave in OF with FBG [16]: (**a**) input; (**b**) reflected; (**c**) transmitted.

The measurement principle of the optical fiber sensor is to use the characteristic that the wavelength of light reflected from each grating depending on changes in external conditions, such as temperature or intensity. As shown in Figure 7, when a wide spectrum is incident, the FBG reflects only a specific wavelength and transmits the other wavelengths. When the ambient temperature of the FBG changes or tension is applied to the grating, the specific wavelength reflected undergoes a change as the reflective index or length of the optical fiber changes. Therefore, temperature, tension, pressure and bending can be detected by measuring the wavelength of light reflected from the FBG. When a wide spectrum is incident on the OF, the reflected signal causes interference and is reflected on the OF grating, and the remaining wavelengths pass through and are not involved in the measurement as shown in Equation (1) [27].

(1) $$\lambda_{B}=2n\Lambda$$

where $\lambda_{B}$ is reflected wave length (μm), n is effective reflection index, Λ is the period of FBG.

The Bragg wavelength reflected from the grating is a function of the effective refractive index and the grating period. The main meaning of the effective refractive index and the grating is a function of the temperature and strain. When temperature or deformation is given to the fiber Bragg grating, the reflected wavelength varies. In the Bragg equation, if the Bragg wavelength is differentiated, the temperature, strain, effective refractive index and grating period are then substituted, and Equation (2) can be obtained as follows.

(2) $$\Delta \lambda_{B}=\lambda_{B}\left[\left(\alpha +\xi \right)\Delta T+\left(1-P_{e}\right)\Delta \varepsilon \right]=K_{T}\Delta T+K_{\varepsilon }\Delta \varepsilon$$

where $\Delta \lambda_{B}$ is the change of wave length (μm), α is expansion coefficient corresponding to temperature, ξ is coefficient considering the change of reflection corresponding to temperature, ΔT is temperature change, $P_{e}$ is photo-elastic coefficient, Δε is the change of strain in FBG, $K_{T}$ and $K_{\varepsilon }$ are coefficients considering the changes of temperature and reflection, respectively.

Therefore, the strain can be calculated from the measured wavelength change information as shown in Equation (3).

(3) $$\Delta \varepsilon =\frac{\Delta \lambda_{B}-K_{T}\Delta T}{K_{\varepsilon }}=\frac{1}{1-P_{e}}\left[\frac{\Delta \lambda_{B}}{\lambda_{B}}-\left(\alpha +\xi \right)\Delta T\right]$$

The photoelastic modulus ($P_{e}$), reflected wave length ($\lambda_{B}$), expansion coefficient corresponding to temperature (α), coefficient considering the change of reflection corresponding to temperature (ξ) values of the OF used in this study are summarized in Table 5.

**Figure 8:**
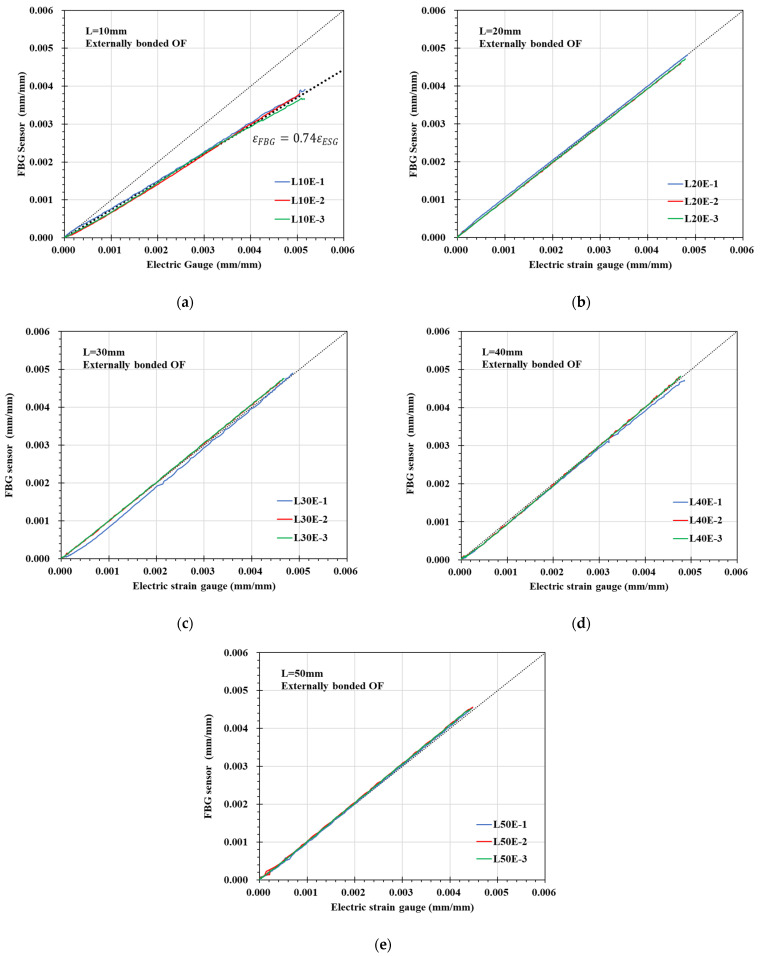
Comparison of strain values of FBG-OF and ESG: (**a**) L10E; (**b**) L20E; (**c**) L30E; (**d**) L40E; (**e**) L50E.

Equation (3) was used to convert the tensile test results of the FBG-OF sensor according to the adhesion length into strain and compare it with the strain information of the ESG as shown in Figure 8. In Figure 8, the diagonal dotted line is the reference line indicating the case where the values of the horizontal and vertical axes are the same. As the applied tensile stress on the specimen increased, an increase in the strain of the ESG and FBG-OF sensor showed a linear relationship for all adhesion lengths. Meanwhile, it was found that the strain of the FBG-OF sensor whose half bond length “L” is 10 mm consistently exhibits a value corresponding to about 74% of the electric gauge strain. This is because when the adhesion length is 10 mm, the strain occurring in the FRP is not sufficiently transferred to the BFG-OF sensor due to the shear lag effect. On the other hand, when the adhesion length was 20 mm or more, the strain of the FBG-OF sensor was almost identical to that of the electric gauge.

## 4. Strain Transfer Mechanism of the Externally Bonded FBG-OF Sensor to FRP Strip

**Figure 9:**
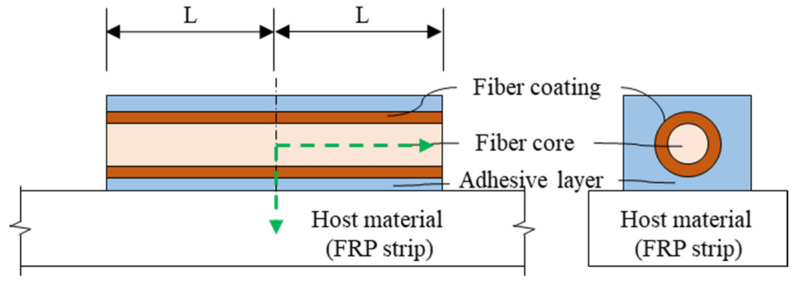
Closed look of FBG-OF sensor bonded on FRP strip with epoxy resin.

**Figure 10:**
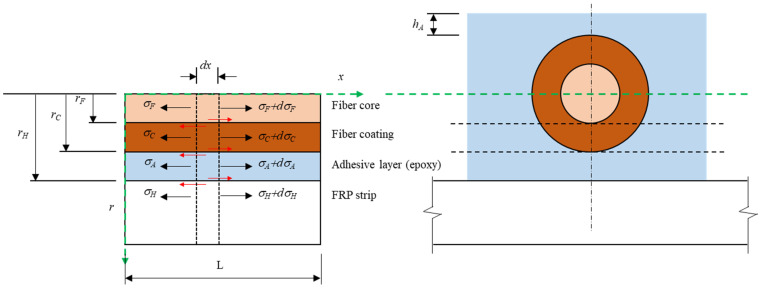
Analytical model of FBG-OF bonded to FRP strip.

The details of the case in which the FBG-OF is attached on the FRP strip as in this study are shown in Figure 9. In this case, a theoretical model describing a relationship in which the deformation generated in the FRP strip is transferred to the FBG-OF sensor is based on the theoretical model of Ansari et al. [6] and Zhao et al. [28], as shown in Figure 10. When tensile stress occurs in the FRP, the deformation caused by the stress is transferred to the FBG-OF sensor through epoxy, resulting in a change in optical signal transfer [29]. The change of strain transition in the FBG-OF attached to the surface of the FRP can be estimated by the following assumptions [28,29,30,31]. The fiber core, coating and FRP strips behave as linear elastic isotropic materials. The FRP exhibits homogeneous behavior along the length of the fiber.

FRP strips are subjected to uniform axial stress, while the fiber core, coating and adhesive layer are not subjected to any external loadings. The interfaces of all materials are fully bonded, and the displacements maintain consistency along the interfaces.

Given the above assumptions and the symmetry in the orthogonal direction, the equilibrium equation for the infinitesimal fiber core is shown below.

(4) $$\pi r_{F}{}^{2}\left(\sigma_{F}+d\sigma_{F}-\sigma_{F}\right)+2\pi r_{F}\tau \left(x,r_{F}\right)dx=0$$

where

(5) $$∴\frac{d\sigma_{F}}{dx}=-\frac{2\tau \left(x,r_{F}\right)}{r_{F}}$$

In a similar manner, the equilibrium equations for the infinitesimal fiber coating and adhesive layer are represented, respectively, as follows.

(6) $$∴\frac{d\sigma_{C}}{dx}=\frac{2\left[r_{F}\tau \left(x,r_{F}\right)-r_{C}\tau \left(x,r_{C}\right)\right]}{r_{C}{}^{2}-r_{F}{}^{2}}$$

(7) $$∴\frac{d\sigma_{A}}{dx}=-\frac{2\pi r_{C}\tau \left(x,r_{C}\right)-W_{A}\tau \left(x,r\right)}{W_{A}\left(h_{A}+r_{C}+r\right)-\pi r_{C}{}^{2}}$$

If Equations (5)–(7) are combined, Equation (8) can be obtained as shown below.

(8) $$∴\tau \left(x,r\right)=-\frac{r_{F}{}^{2}}{W_{A}}\frac{d\sigma_{F}}{dx}-\frac{\pi \left(r_{C}{}^{2}-r_{F}{}^{2}\right)}{W_{A}}\frac{d\sigma_{C}}{dx}-\left(h_{A}+r_{C}+r-\frac{\pi r_{C}{}^{2}}{W_{A}}\right)\frac{d\sigma_{A}}{dx}$$

The stress transfer between the FRP and the FBG-OF is mostly dominated by the shear modulus of elasticity, followed by the radial deformation of OF. Therefore, the Poisson effect can be ignored in this case. Equation (9) can be derived by substituting σ=Eε, ε=du/dx into Equation (8).

(9) $$∴\tau \left(x,r\right)=-\frac{\pi r_{F}{}^{2}E_{F}}{W_{A}}\frac{d\varepsilon_{F}}{dx}-\frac{\pi \left(r_{C}{}^{2}-r_{F}{}^{2}\right)E_{C}}{W_{A}}\frac{d\varepsilon_{C}}{dx}-\left(h_{A}+r_{C}+r-\frac{\pi r_{C}{}^{2}}{W_{A}}\right)E_{A}\frac{d\varepsilon_{A}}{dx}$$

Because the fiber core is deformed as other layers are synthetized, the deformations of all layers are almost the same.

(10) $$\frac{d\varepsilon_{C}}{dx}≅\frac{d\varepsilon_{A}}{dx}≅\frac{d\varepsilon_{F}}{dx}$$

Equation (11) can be obtained by substituting Equation (10) into Equation (9), as follows.

(11) $$\tau \left(x,r\right)=\left[-\frac{\pi r_{F}{}^{2}}{W_{A}}E_{F}-\frac{\pi \left(r_{C}{}^{2}-r_{F}{}^{2}\right)}{W_{A}}E_{C}-\left(h_{A}+r_{C}+r-\frac{\pi r_{C}{}^{2}}{W_{A}}\right)E_{A}\right]\frac{d\varepsilon_{F}}{dx}$$

Since the FBG sensor exhibits a long aspect ratio, the radial displacement can be ignored. That is,

(12) $$\tau \left(x,r\right)=G_{A}\gamma \left(x,r\right)=G_{A}\gamma \left(\frac{\partial u}{\partial r}+\frac{\partial w}{\partial x}\right)≅G_{A}\frac{\partial u}{\partial r}$$

If Equation (12) is substituted into Equation (11) and then integrated with $\left(r_{F},r_{H}\right)$,

(13) $$\int_{r_{F}}^{r_{H}}\left(G_{A}\frac{du}{dr}\right)dr=-\int_{r_{F}}^{r_{H}}\left[\frac{\pi r_{F}{}^{2}}{W_{A}}E_{F}+\frac{\pi \left(r_{C}{}^{2}-r_{F}{}^{2}\right)}{W_{A}}E_{C}+\left(h_{A}+r_{C}+r-\frac{\pi r_{C}{}^{2}}{W_{A}}\right)E_{A}\right]dr$$

(14) $$u_{H}-u_{F}=-\frac{r_{H}-r_{F}}{W_{A}-G_{A}}\left\{\pi r_{F}{}^{2}E_{F}+\pi \left(r_{C}{}^{2}-r_{F}{}^{2}\right)E_{C}+\left[W_{A}\left(h_{A}+r_{C}+\frac{r_{F}}{2}+\frac{r_{H}}{2}\right)-\pi r_{C}{}^{2}\right]E_{A}\right\}\frac{d\varepsilon_{F}}{dx}\;=-\frac{1}{k^{2}}\frac{d\varepsilon_{F}}{dx}$$

We can obtain;

(15) $$k=\sqrt{\frac{W_{A}G_{A}}{\left(r_{H}-r_{F}\right)\left\{\pi r_{F}{}^{2}\cdot E_{F}+\pi \left(r_{C}{}^{2}-r_{F}{}^{2}\right)\cdot E_{C}+\left[W_{A}\left(h_{A}+r_{C}+\frac{r_{F}}{2}+\frac{r_{H}}{2}\right)-\pi r_{C}{}^{2}\right]\cdot E_{A}\right\}}}$$

where k is the strain lag parameter reflecting the effects of the fiber core, coating and adhesive layer. Differentiating Equation (14) with respect to x, yields:

(16) $$\frac{d^{2}\varepsilon_{F}\left(x\right)}{dx^{2}}-k^{2}\varepsilon_{F}\left(x\right)=-k^{2}\varepsilon_{H}$$

The general solution of Equation (16) is

(17) $$\varepsilon_{F}\left(x\right)=C_{1}e^{kx}+C_{2}e^{-kx}+\varepsilon_{H}$$

$C_{1}$ and $C_{2}$ are the integration constants and obtained by considering the boundary condition that the axial stress of the fiber core is assumed free at the both ends.

(18) $$\varepsilon_{F}\left(L_{F}\right)=-\varepsilon_{F}\left(L_{F}\right)=0$$

(19) $$C_{1}=C_{2}=\frac{\varepsilon_{H}}{2\;\cos h\left(k\cdot L_{F}\right)}$$

Thus, the strain relationship of the fiber core to the host material at a given x coordinate is

(20) $$\varepsilon_{F}\left(x\right)=\varepsilon_{H}\left(1-\frac{\cos h\left(k\cdot x\right)}{\cos h\left(k\cdot L_{F}\right)}\right)$$

The maximum strain transfer rate $\psi_{H}\left(0\right)$ happens at the midpoint of the fiber core (i.e., the point where x is equal to zero), and yields to:

(21) $$\psi_{H}\left(0\right)=\frac{\varepsilon_{F}\left(0\right)}{\varepsilon_{H}}=1-\frac{1}{\cos h\left(k\cdot L_{F}\right)}$$

Average strain transfer rate $\psi_{avg}$ can be expressed as

(22) $$\psi_{avg}=\frac{\bar{\varepsilon_{F}\left(x\right)}}{\varepsilon_{H}}=\frac{2\int_{0}^{L}\varepsilon_{F}\left(x\right)dx}{2L_{F}\cdot \varepsilon_{H}}=1-\frac{\sin h\left(k\cdot L_{F}\right)}{k\cdot L_{F}\cos h\left(k\cdot L_{F}\right)}$$

## 5. Evaluation of the Sensing Capacity of the FBG-OF Sensor Externally Bonded to FRP Strip

The strain transition between the FBG-OF and FRP of specimens shown in Table 1 was evaluated in accordance with the aforementioned strain transfer mechanism. The material properties of OF, epoxy and FRP are summarized in Table 6. These values are the information provided by the manufacturer and the values used by the previous researchers [28,31]. The cross-sectional dimensions of materials applied in the theoretical analysis are shown in Table 7.

**Figure 11:**
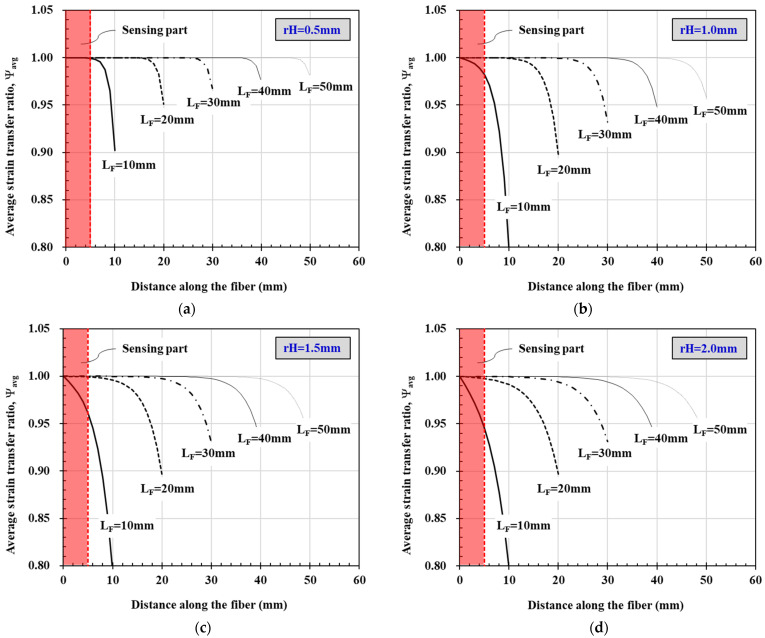
Average strain transfer ratio: (**a**) *r_H_* = 0.5 mm; (**b**) *r_H_* = 1.0 mm; (**c**) *r_H_* = 1.5 mm; (**d**) *r_H_* = 2.0 mm.

Figure 11 shows changes in the average strain transfer rate ψ_avg_ according to the adhesion length of the FBG-OF sensor evaluated using the analysis model. As the adhesion length, *L_F_*, is changed from 10 mm to 50 mm, the strain transfer rate and the strain ratio of the FBG-OF to FRP decrease rapidly from each adhesion length to the end portion. In addition, as the adhesion length increases, the section in which a reduction in the strain transfer rate does not occur gradually increases. In the figure, the shaded part is the section, which is up to 5 mm from the center, where the FBG is formed in the OF, thus enabling actual sensing. As the thickness (*r_H_*) of the epoxy resin, which is the adhesive, increases, the average transfer rate decreases. When *r_H_* is 0.5 mm, the average strain transfer ratio at the sensing area does not decrease even when L_F_ is 10 mm. However, when the thickness is larger than that, a decrease occurs. When *r_H_* is 1.5 mm, *L_F_* must be 20 mm or more so that a decrease in the average strain transfer ratio does not occur at the sensing area. On the other hand, when *r_H_* is 2.0 mm, the average strain transfer ratio does not satisfy the required at the sensing area even if *L_F_* is 20 mm or more.

Meanwhile, in all cases, when *L_F_* is 10 mm, the transfer ratio decreases rapidly within a sensing part of 5 mm, and the strain ratio of OF to FRP also drops sharply. Therefore, when *L_F_* is 10 mm, the strain that occurs in the FRP is not sufficiently transferred to the OF due to the shear lag phenomenon. On the other hand, when *L_F_* is 20 mm or more, there is almost no change in the average strain transfer, and a decrease in the in the strain ratio, *ε_F_/ε_H_* rarely occurs within the sensing part.

**Figure 12:**
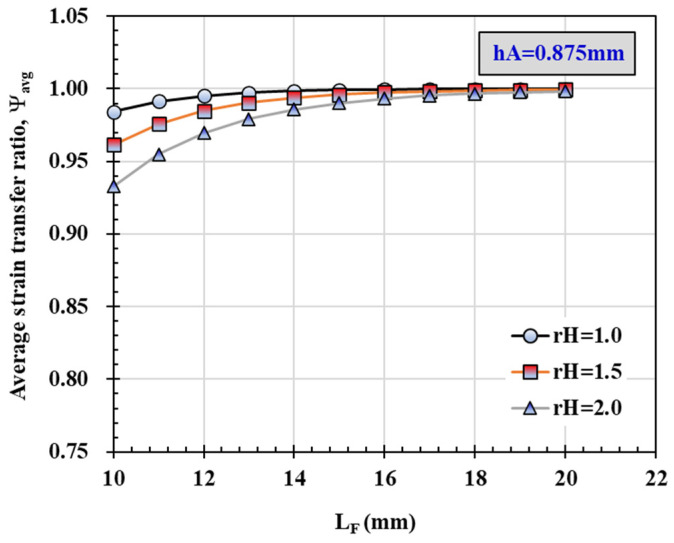
Average strain transfer ratio at a distance of 5 mm from the center corresponding to the change of epoxy thickness between OF and FRP, *r_H_*.

**Figure 13:**
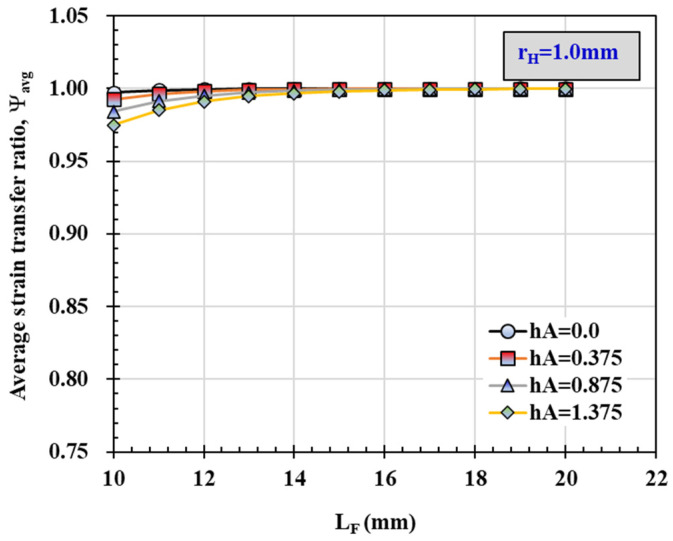
Average strain transfer ratio at a distance of 5 mm from the center corresponding to the change of cover thickness of epoxy resign, *h_A_*.

Figure 12 shows the average strain transfer ratio in the sensing part according to the *L_F_* change at *r_H_* 1.0, 1.5 and 2.0 when *h_A_* is 0.875mm. In addition, when *r_H_* is 1.0mm, changes in the average strain transfer ratio according to the *L_F_* change at *h_A_* 0, 0.375 and 1.375 are shown in Figure 13. It can be confirmed that as *r_H_* and *h_A_* increase, the average strain transfer ratio decreases, and the difference is very large when the *L_F_* value is small. In addition, the effect of *h_A_* was found to be greater than that of *r_H_*.

Given the error in the thickness of epoxy, the strain generated in the FRP can be sufficiently transferred to the FBG-OF sensor when *L_F_* is 20 mm or more. From this, it can be confirmed that the values calculated using the model are very similar to the test results. Therefore, it is expected that the proposed model equation can be used to properly predict the strain transfer of FBG-OF sensor attached to the surface of FRP, and it can also be utilized in the determination of the optimal adhesion length.

## 6. Conclusions

This paper sought to determine the appropriate adhesion length when an FBG-based OF sensor is externally bonded to an FRP strip used for the reinforcement of concrete members with an epoxy resin. Toward this end, an OF with FBG was externally attached to the surface of an FRP strip, and an experiment to evaluate the sensing performance was then conducted with the adhesion length as a variable. In addition, an analysis model was applied to evaluate the strain transfer ratio according to the adhesion length.

The tensile test on the FRP strip with externally bonded FBG-OF found that as the tensile strain increases, an increase in the strain of the electric gauge and OF sensor shows a linear relationship for all adhesion lengths. It was also found that the strain of OF sensor with a half bond length of 10 mm consistently exhibits a value corresponding to about 74% of the electric gauge strain because the strain is not sufficiently transferred to the OF sensor due to the shear lag effect. On the other hand, when the adhesion length was 20 mm or more, the strain of the OF sensor was almost identical to that of the electric gauge as the strain of FRP was sufficiently transferred to the OF sensor.

Based on the theoretical models, a model of the case in which the OF sensor is bonded to the surface of the FRP strip with epoxy was built, and the sensing performance according to the adhesion length was evaluated. The results showed that as the adhesion length for the OF sensor, *L_F_*, was changed from 10 mm to 50 mm, the strain transfer rate and the strain ratio of OF sensor to FRP tended to decrease rapidly from each bonding area to the end portion. In addition, the section in which a reduction in the strain transfer rate does not occur increased gradually with increasing adhesion length. In particular, the average strain transfer ratio decreased as the thickness of epoxy between FRP and OF, and the thickness of epoxy applied to the OF increased, and the effect of the epoxy thickness between FRP and OF was found to be greater among the two factors.

The analysis results suggest that given the error in the thickness of epoxy when the actual FBG-OF sensor is bonded to the FRP strip, the strain generated in the FRP can be sufficiently transferred to the OF sensor if the OF is bonded with epoxy so that the half bond length, *L_F_*, can be more than 20 mm, that is, the total length is 40 mm or more, which are very similar to the test results. From this, it is expected that the model can be used to properly predict the strain transfer of an FBG-OF sensor attached to the surface of FRP with epoxy, and it can also be utilized in the determination of the optimum adhesion length.

## Figures and Tables

**Table 1:** Specimen list.

Specimen Name	Adhesive	Adhesive Length, L (mm)	$\mathrm{Adhesive}\;\mathrm{Width},\;W_{A}$ (mm)	$\mathrm{Adhesive}\;\mathrm{Thickness},\;t_{e}$ (mm)
L10E	Epoxy resign	10	10	2
L20E		20		
L30E		30		
L40E		40		
L50E		50		

**Table 2:** Dimension and tensile strength of optic fiber.

Type	Diameter of Core (mm)	Diameter (mm)	Tensile Strength (N/mm^2^)
FBG-bare-310	0.125	0.250	690
Data was given by the manufacturer			

**Table 3:** Dimension and mechanical properties of FRP strip.

Type	Thickness (mm)	Width (mm)	Tensile Strength (N/mm^2^)	Elastic Module (N/mm^2^)
SK-CPS 0512 (Carbon fiber)	1.2	50	2942	165,000
Data was given by the manufacturer				

**Table 4:** Mechanical properties of epoxy.

Type	Compressive Strength (N/mm^2^)	Shear Bond Strength (N/mm^2^)	Bond Strength to Concrete (N/mm^2^)
SK-CPA10	90	10	1.5
Data was given by the manufacturer			

**Table 5:** Optical properties of FBG-OF.

Material	$P_{e}$	$\lambda_{B}$ (μm)	α ($\times 10^{-6}$/°C)	ξ ($\times 10^{-6}$/°C)
Silica fiber	0.22 *	1050 ^+^	0.55 *	8.6 *

* These are the values used in the research by Werneck et al. [27]. ^+^ Value suggested by the manufacturer.

**Table 6:** Mechanical properties for OF, epoxy and FRP considered in the model.

Material		Value
Fiber core	Young’s modulus, $E_{F}\;\left(P_{a}\right)$	$7.2\times 10^{10}$
	Poisson’s ratio, $\lambda_{F}$	0.17
Fiber coating	Young’s modulus, $E_{C}\;\;\left(P_{a}\right)$	$2.55\times 10^{6}$
	Poisson’s ratio, $\lambda_{C}$	0.48
Epoxy	Young’s modulus, $E_{A}\;\left(P_{a}\right)$	$3.4\times 10^{9}$
	Poisson’s ratio, $\lambda_{A}$	0.34
	Shear modulus, $G_{A}\;\left(P_{a}\right)$	$1.27\times 10^{9}$
FRP	Young’s modulus, $E_{H}\;\left(P_{a}\right)$	$1.65\times 10^{8}$
	Poisson’s ratio, $\lambda_{H}$	0.35

**Table 7:** Dimensions of OF, epoxy and FRP in the model.

Material	Value (mm)
Half diameter of fiber core, $r_{F}$	0.0625
Half diameter of fiber coating, $r_{C}\;$	0.125
Half thickness of epoxy, $r_{H}\;$	1.0
Bonded width of epoxy, $W_{A}\;$	10
Thickness of FRP,$\;W_{H}\;$	2.4

## Data Availability

Data sharing is not applicable to this article.
